# Impacts of Different Perinatal Factors on Faecal Immune Compounds in Infants: Determination of Normal Values

**DOI:** 10.3390/ijms251910675

**Published:** 2024-10-03

**Authors:** Marta Suárez, Gonzalo Solís, Laura Mantecón, Miguel Gueimonde, Silvia Arboleya

**Affiliations:** 1 Pediatrics Service, Central University Hospital of Asturias (HUCA-SESPA), 33011 Oviedo, Spain; msr1070@hotmail.com (M.S.); solisgonzalo@uniovi.es (G.S.); laura_mantecon@hotmail.com (L.M.); 2 Institute of Health Research of the Principality of Asturias (ISPA), 33011 Oviedo, Spain; 3 Department of Medicine, Faculty of Medicine and Health Sciences, Universidad de Oviedo, 33011 Oviedo, Spain; 4 Primary Care Interventions to Prevent Maternal and Child Chronic Diseases of Perinatal and Developmental Origin Network (RICORS), 33011 Oviedo, Spain; 5 Department of Microbiology and Biochemistry of Dairy Products, Instituto de Productos Lácteos de Asturias (IPLA-CSIC), 33300 Villaviciosa, Spain

**Keywords:** immune compounds, immunoglobulins, cytokines, faeces, gut microbiota, infants, health

## Abstract

The gut microbiota is a key and primary stimulus for the development of a host’s immune system. The early establishment of the gut microbiota is affected by several perinatal factors but little is known about their influence on shaping normal immune development and, consequently, on the programming of future health. The analysis of different immune compounds is well-documented in serum samples; however, their presence in faecal samples has not been studied, and this information could be valuable in early life. In this context, the authors of this study aimed to both describe the immunological faecal profile of a cohort of one-month-old infants and describe the impact of different perinatal factors, exploring possible associations between immune compounds and gut microbiota in faecal samples. Clear differences in immune profile were observed between full-term and premature infants. Breastfeeding increases IgG2, IgG4, and IgA; in addition, male babies showed some increased Igs, among other observations. Overall, the findings of this study reinforce the hypothesis that microorganisms and immune compounds interact with each other in the early neonatal gut and that understanding these interactions in depth will help us comprehend the influence of the gut microbiota on short- and long-term infant health outcomes.

## 1. Introduction

The gut microbiota, which harbours a complex and dynamic bacterial community, is a major microbial stimulus and provides a primary signal for the development of a host’s innate and adaptive immune system; meanwhile, the immune system coordinates host–microbe symbiosis [1]. The delayed acquisition of, or alterations in, the gut microbiota may delay the maturation of the adaptive immune system, resulting in irreversible systemic effects on host health [2]. Thus, the early establishment of the gut microbiota is a continuous process influenced by several perinatal factors, such as gestational age, delivery mode, feeding regiment, and antibiotic treatment [3]. These factors can influence normal immune mucosal and systemic development.

Although several key steps in the development of the immune system occur in utero, there are many others that need postnatal antigen exposure. During delivery, the newborn infant emerges from the sterile maternal intrauterine environment, and the infant’s inexperienced immune system must adapt to a new world populated with a massive collection of microorganisms, some of which are friends and some of which are foes. In the early neonatal period, the infant profits from a hybrid maternal–infant immune system, and even though the neonate’s innate immune system can generate an immediate response against potential pathogens immediately after birth, it needs to mature in coordination with the microbiota and maternal and environmental factors [4,5].

Some perinatal factors affecting the establishment of the gut microbiota [3] are also involved in the development of the immune system. Different animal studies have underscored that the development of the regulatory immune system is affected by the delivery mode [2,6,7]. Observational human studies have also shown that there is an increased risk of developing childhood asthma or other immune-related disorders after caesarean delivery [8,9]. Several studies have demonstrated the impact of antibiotics not only on the secretion of different cytokines and chemokines but also on the modulation of the expression of Toll-like receptors and the regulation of monocyte phagocytic activity [10]. The antibiotic-impacted microbiota can generate different immune cells in the colon, thus resulting in long-lasting damage and jeopardizing the infant’s ability to deactivate allergic responses [11]. Breastfeeding is another factor affecting the development of the immune system. One human study observed that neonatal immune tolerance was promoted by breast milk in response to antigenic stimulation by increasing the proportion of Treg cells and reducing the proliferation of T helper cells and cytokine production [12]. Different milk molecules drive both innate and adaptive immune maturation. IgA present in breast milk has an important impact on NEC prevention by binding to intestinal bacteria [13]. Animal studies have shown how IL-7 in breast milk correlates with thymic development in neonate offspring, and TGF-β in breast milk stimulates neonatal mucosal IgA production and inhibits the synthesis of pro-inflammatory cytokines [14]. By comparing the immune profiles in meconium and faecal samples obtained at 2-year follow-up in premature babies, an observational human study has also shown different immune maturation of the gut, in parallel with gut microbiota development [15]; however, the effect of prematurity per se has not yet been studied.

Different immune compounds, such as immunoglobulins and cytokines, can be detected in faecal samples from infants in their first weeks of life. Immunoglobulins (Igs) are glycoproteins produced by B cells, and, among the different types, IgA is the primary mediator of humoral mucosal immunity and has the highest amount of secretion in the intestinal tract [16,17]. However, in contrast to IgG and IgM levels, the generation of IgA is limited during early infancy, and the delayed development of mucosal IgA production might lead to infectious disease in young infants [18]. Cytokines are key regulators of the inflammatory response, orchestrating both pro-inflammatory (interleukin (IL)-1, IL-6, IL-8, TNF, and IFN-γ) and anti-inflammatory (e.g., IL-10, IL-4, and IL-5) effects and the maturation of various immune cells [19]. The detection and concentration of some of these immune compounds are well-documented in serum samples both in infant and adult populations [20,21,22]. However, only a few studies have provided information about the concentration of Ig and IL in infant faeces, and it is always presented in a disease framework [15,23,24].

There is important evidence that the initial microbial intestinal colonization and the subsequent immune and metabolic programming could have a lasting influence on the risk of future diseases, in line with the theory of the developmental origins of health and disease [25,26]. However, little is known about the possible influence of this early gut microbiota colonization on the intestinal immune environment and, consequently, on the programming of future health. Immune profiling in infant faeces could provide valuable information about the composition and function of the early development of the immune system.

In this context, this study aimed to both describe the immunological faecal profile of a cohort of one-month-old infants and describe the impact of different perinatal factors, thus exploring possible associations between immune compounds and gut microbiota in faecal samples.

## 2. Results

The immunological profile—including six immunoglobulin types and nine cytokines analysed using magnetic bead-based multiplex immunoassays—was characterized in 67 faecal supernatant samples from one-month-old infants. Different prevalences and abundances were observed for the immune compounds analysed. The complete set of immunoglobulins was present in fifteen samples, and ten infants were also positive in the prevalence and quantification for the nine cytokines. The rest of the samples showed different occurrences for each immune compound analysed. Among the immunoglobulins, IgG2 was quantified in 100%, IgA in 97%, and IgG1 and IgG4 in 85% and 84% of infants, respectively. As expected, IgA showed the highest concentration in faeces (expressed as median (IQR)), 2.66 (0.2–8.19) mg/g, followed by IgG2 and IgM displaying concentrations of 29.16 (13.62–70.9) µg/g and 20.85 (6.27–53.15) µg/g of faeces, respectively. The occurrence rates for cytokines ranged from 48% of babies for IL12 to 18% of babies for IL2, IL 10, IL13, and TNF-α, with concentrations of (expressed as the median) 3.97, 14.40, 17.10, 5.60, and 40.20 pg/g of faeces, respectively. Other than these general observations, differences in the occurrence and concentration of the different faecal immune compounds were observed in the infant population under different perinatal factors as it is depicted below.

### 2.1. Faecal Immune Profile Varies with Gestational Age

**Figure 1:**
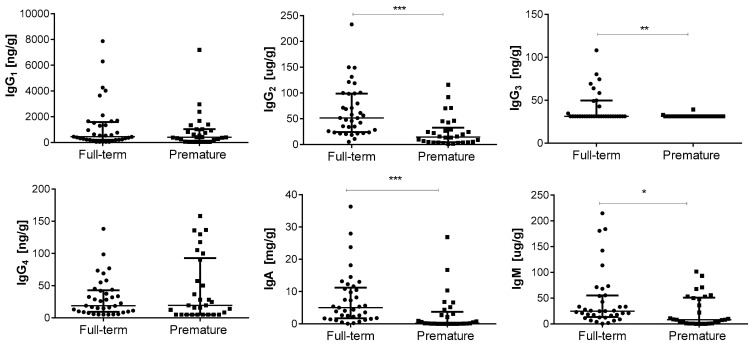
Concentration (median—IQR) of immunoglobulins analysed in faecal samples from one-month-old full-term (*n* = 37) and premature (*n* = 30) infants. * *p* < 0.05; ** *p* < 0.01; *** *p* < 0.001. IQR: interquartile range.

Gestational age is known to have an important impact on the physiology and homeostasis of infants and even on the establishment of the gut microbiota. Therefore, the impact of prematurity was analysed on the faecal immune compound load. Full-term babies showed an increased occurrence of the whole set of immunoglobulins with respect to premature babies, with the sole exception of IgG2, which was quantified in all the samples belonging to both infant groups. The higher prevalences of IgG3 and IgG4 in full-term faeces were statistically significant (*p* < 0.01) (Table 1). In addition, the concentration of IgG2, IgG3, IgA, and IgM was also significantly higher in samples from the full-term group than in samples from preterm infants (Figure 1). While the concentration of IgG2 and IgM in full-term faeces trebled the one in premature faeces, the concentration of IgA increased by almost a factor of fourteen. In contrast, no differences were observed in IgG1 and IgG4 concentrations between both groups of infants.

All of the cytokines also occurred at different quantification frequencies between full-term and premature samples. Faeces of full-term infants only showed presence at quantifiable levels of IL12 in 37.8% of the samples, IL4 in 24.3% of the samples, and IL5 in only one sample (2.7%) at a low concentration (59.73 pg/g). Meanwhile, premature babies presented a statistically (*p* < 0.01) higher prevalence in the complete set of these immune compounds, with the sole exception for IL12 (*p* = 0.071). Only IL4 and IL12 reached 60% of prevalence in the complete set of samples (Table 1). The concentration of cytokines detected in premature faeces is higher (*p* < 0.001) than that in the full-term group (Table 2).

The large differences observed between the premature and full-term groups of infants precluded us from combining them for further analyses, and consequently, they were hereafter considered two independent groups.

### 2.2. Effect of Different Feeding Regimens on Faecal Immune Profile

The full-term infants enrolled in this study had different feeding regimes (exclusively breast-fed, formula-fed, or mixed-fed), and differences in the immune compounds were observed. IgG2, IgA, and IgM were present in 100% of the samples, followed by IgG4 (94.59% prevalence), IgG1 (89.19% prevalence), and IgG3 (35.14% prevalence) but no statistical differences were observed based on the feeding regimen. However, breast milk-fed infants faeces showed higher concentrations of IgG2 (*p* < 0.01), IgG4 (*p* < 0.05), and IgA (*p* < 0.01) (Table 3). On the other hand, IL 4 and IL12 showed higher quantification frequencies in formula-fed than breast-fed and mixed-fed infants (n.s) (44% and 55% occurrence, respectively) (Table S1).

The complete set of Igs was quantified in more than 80% of premature faeces, except for IgG4, which was less prevalent (*p* < 0.05) in formula-fed infants, and IgG3 was only present in 11% of mixed-fed babies (Table S1). However, none of the premature babies were exclusively fed with breast milk at one month of age, and mixed-fed infants showed higher levels of immunoglobulins than exclusively formula-fed premature infants. IgG2, IgG4, and IgA reached significantly higher concentrations (*p* < 0.05, *p* < 0.05, and *p* < 0.01, respectively) (Table 3). In contrast, formula feeding showed a trend of increasing the levels and quantification frequencies of cytokines (Table S1).

### 2.3. Faecal Immune Profile Is Affected by Intrapartum Antimicrobial Prophylaxis

Faecal samples from full-term babies whose mother received intrapartum antibiotics showed a significant increment in the concentration of IgG1 (*p* < 0.05) and a trend towards increased IgG4 (*p* = 0.061) (Table S2). Similarly, IL4 and IL12 also showed a trend of increase in their occurrences (*p* = 0.088, *p* = 0.075, respectively) in full-term IAP babies’ faecal water (42% and 58%, respectively) with respect to premature babies (16% and 28%, respectively). On the contrary, premature babies did not show any differences in the prevalence and quantification of any immune compounds based on IAP treatment (Table S2).

### 2.4. Effects of Other Perinatal Factors on Faecal Immune Profiles: Gender, Antibiotics, and Delivery Mode

**Figure 2:**
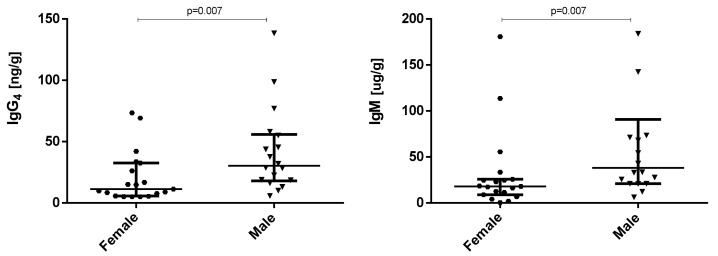
Concentration (median—IQR) of immunoglobulins showing significant (*p* < 0.05) differences (IgG4 and IgM) between faecal samples from one-month-old full-term females (*n* = 19) and males (*n* = 18).

When comparing other factors, such as gender, we observed statistical differences in the concentration of IgG4 and IgM in full-term infants (*p* < 0.01). While female babies showed concentrations of 11.3 ng/g and 17.91 µg/g, respectively, male babies displayed concentrations of about 30.35 ng/g and 38.10 µg/g, respectively. No statistical differences were observed in premature babies (Figure 2; Table S3).

**Figure 3:**
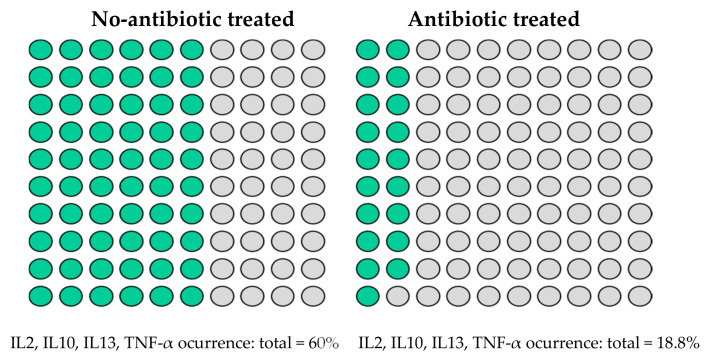
Occurrence (%) of cytokines showing significant (*p* < 0.05) differences (IL2, IL10, IL13, and TNF-α) in faecal samples between one-month-old premature no-antibiotic-treated (*n* = 10) and antibiotic-treated (*n* = 16) infants.

On the other hand, the premature population had a 73.3% rate of C-section deliveries, and 61.5% of the infants were administered antibiotics. When studying the effect of delivery mode on the immune compound load, no statistically significant differences in immunoglobulin prevalence and concentration were observed; however, it is interesting to note that there was a higher IgA concentration in vaginally delivered premature babies (0.69 mg/g) compared with those delivered via C-section (0.19 mg/g). With respect to antibiotic use, cytokines were quantified in more samples of premature infants who were not administered antibiotics, and this was significant for IL2, IL10, IL13, and TNF-α (*p* < 0.05) (Figure 3).

### 2.5. Global Comparison of Immunological and Microbial Profiles in Faecal Samples from Full-Term and Preterm Infants

Noticeable differences in the development of the composition of the intestinal microbiota between full-term and preterm babies have been repeatedly reported. At the age of 1 month, the gut microbiota of the full-term babies enrolled in this study were dominated by four main phyla: Actinomycetota (38% relative abundance), Pseudomonadota (30%), Bacillota (23%), and Bacteroidota (7%); while at the genus level, *Bifidobacterium*, *Escherichia-Shigella*, *Streptococcus*, *Vellionella*, and *Bacteorides* are the most abundant. On the other hand, the gut microbiota of the premature baby group are dominated by Pseudomonadota (70% relative abundance) followed by Bacillota (23%). The most abundant genera are *Escherichia-Shigella*, *Streptococcus*, and *Klebsiella* (Figure S1). The differences in the composition of the gut microbiota are reflected in the main microbial metabolites, short-chain fatty acids. The concentrations of acetate and propionate in full-term infant faeces are higher than those in premature babies; however, the premature group depicted higher levels of butyrate (Figure S2).

**Figure 4:**
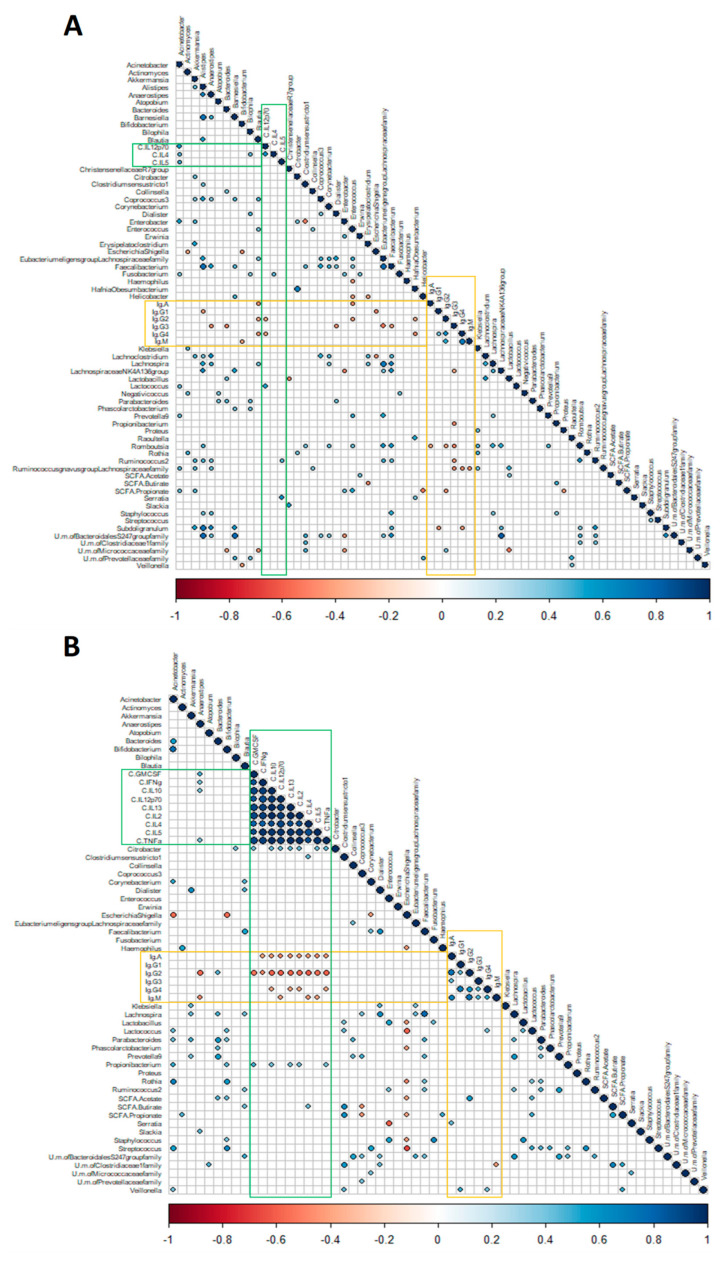
Corrplot showing significant (*p* < 0.05) Spearman correlation coefficients between relative abundances of microbiota at genus level, SCFAs, and immune compounds in one-month-old (**A**) full-term infants and (**B**) premature infants. Blue and red colours denote positive and negative associations. The intensity of the colours and the areas of circles or squares show the absolute values of corresponding correlation coefficients. Only significant associations (*p* < 0.05) are represented. Significate results between immune compounds and microbiota or SCFAs are highlighted in green for cytokines and yellow for immunoglobulins.

An exploration into the association of the concentration of immune compounds with faecal microbiota composition at the taxonomic genus level was conducted by using Spearman’s correlation analyses in full-term and premature babies independently. The full-term group showed a greater number of significant associations between immunoglobulins and microbial genera compared to the premature group (Figure 4), and interestingly, all of them were negative (Figure 4A). IgG3 is the immunoglobulin that showed the greatest number of correlations with more genera, notably including *Bacteroides*, *Romboustia*, and *Eubacterium eligens* (*p* < 0.01) followed by IgG2, which was negatively correlated with *Enterococcus* and *Romboustia* (*p* < 0.05), among others, and propionate (*p* < 0.01). IgA, which had the highest concentration in faeces, revealed a negative association with *Blautia*, *Enterococcus*, and *Romboustia* (*p* < 0.05) and IgM with *Bifidobacterium* and *Ruminococcus gnavus* (*p* < 0.05). IgG1 was negatively correlated with *Escherichia-Shigella* (*p* < 0.05), among others (Figure 4A). By contrast, in the premature infant faeces, correlations between *Bacteroides*, *Lactococcus*, *Serratia*, *Vellionella*, and *Anaerostipes* evidenced significant positive associations with IgG2, IgG4, IgA, IgG4, and IgM (*p* < 0.05), respectively, and acetate with IgG2 and IgG4 (*p* < 0.01, *p* < 0.05) (Figure 4B).

Full-term infants evidenced significant positive correlations between IL-4, IL-5, IL-12, and some minority genera (Figure 4A). Meanwhile, *Anaerostipes*, *Citrobacter*, and *Propionibacterium* were directly correlated with most of the interleukins studied in the premature group (Figure 4B). Correlations between SCFAs and interleukins evidenced the opposite behaviour in the latter group; while acetate showed negative associations with most interleukins, positive correlations were found between propionate and butyrate and IL-4, IL-5, IL-12, and GM-CSF (*p* < 0.05). Interestingly, butyrate showed the most significant direct correlation with IL-4 (*p* < 0.01) (Figure 4B).

## 3. Discussion

The exact composition of immune cells in the healthy infant gut remains poorly defined due to the difficulty of obtaining intestinal tissues for research [4]. As with the study of the gut microbiota, faecal materials can represent an accessible type of sample for studying immune compounds derived from the immune cells in the gut. They can provide valuable information about the composition and function of the immune system, especially in the early stages of life when the immature immune system is developing. Some studies have presented data on immune compounds in infant stools but always in a disease framework. Undoubtedly, the interaction between the gut microbiota and immune cells is a key player in the maturation of the immune system, and the factors involved in shaping the early gut microbiota can influence the derived immune compounds.

In this context, the need arises to define the normal values of different immune compounds in neonates’ faecal samples in a healthy condition. To the best of our knowledge, this was the first study to assess different immune factors, including immunoglobulins and cytokines related to Th1/Th2 balance, in stools from one-month-old infants to describe their immunological profiles, and this study evaluated the effect of different perinatal factors involved in the development of the gut microbiota, thus exploring associations between immune compounds and the microbiota.

As expected, our results showed significant differences in the concentration of immunoglobulins and cytokines between full-term and premature infants. The levels of IgG2, IgG3, IgM, and IgA were significantly higher in full-term infants. IgA is a primary mediator of humoral mucosal immunity [16]. The natural massive arrival of bacteria and other antigens to the gut after birth during the correct establishment of intestinal microbiota in full-term infants likely results in a higher concentration of IgA since gut colonization triggers IgA production via the gut-associated lymphoid tissue (GALT) [27]. The frequency of occurrence of the immunoglobulins set in premature babies was around 80–100% for IgG1, IgG2, and IgM, thus being higher than that observed by Gómez et al. in a premature cohort of 21-day-old babies [15]. However, the concentrations in our group of babies were lower than the ones presented in the above-mentioned work [15], thus suggesting some potential methodological biases. On the other hand, the levels of some cytokines, such as IL-2, IL-10, and IL-5, were within the range presented by Gomez et al. When comparing our cohort of full-term and premature babies, the latter group exhibited a higher cytokine concentration, such as TNF-α, whose high concentrations have been reported in children with inflammatory bowel disease and cow’s milk protein allergy [23]. This may suggest that premature babies have guts with a more pro-inflammatory nature, which may be linked to the increased presence of pro-inflammatory microorganisms such as enterobacteria.

IgA was the immune compound that appeared in the highest concentration; however, in the first few weeks of life, infants’ ability to produce IgA is not fully mature, and IgA can be transferred through passive immunity via breastfeeding [28]. The observed differences in IgA concentration between breast-fed infants and formula-fed infants were the highest among the full set of analysed Igs. Breast-fed infants showed higher levels than formula-fed infants among full-term babies, and this observation also held true for premature mixed-fed babies with respect to those exclusively formula-fed. This consistently lower concentration in formula-fed infants correlates with other observations made during the first year of life [29,30,31]. Full-term breast-fed infants and premature mixed-fed infants also showed higher concentrations of IgG2 and IgG4 than formula-fed babies, while formula-fed infants showed an increased cytokine load.

The use of antibiotics in the neonatal period impedes the correct building of microbiota and may lay the foundation for pathological colonization. Consequently, the maturation of the immune system is affected due to its indispensable crosstalk with microbiota. In this cohort of babies analysed, we did not observe differences in the immune compound load from the premature faeces of infants whose mother was administered intrapartum antimicrobial prophylaxis; however, when neonates directly received antibiotics, there was a lower frequency of occurrence of IL2, IL10, IL13, and TNF-α. IL 10 and IL 13 are known to repress the production of pro-inflammatory cytokines via activated macrophages, and with the regulator effect of IL2 of lymphocyte growth and differentiation and Th1/Th2 differentiation [32], this may explain the lower levels in the no-antibiotic-treated infants. On the other hand, full-term IAP-infants exhibited an increase in IL12, which can exert important pro-inflammatory functions.

The delivery mode did not produce any statistically significant effect on the immune compound load in our study cohort; however, higher concentrations of IgA were observed in vaginally delivered premature babies compared to their C-section counterparts, which is in accordance with previous observations by other authors [31]. Stokholm and colleagues [9] addressed different immune parameters in children who had a retained C-section microbiota profile at the age of one year, with low levels of TNFα, IL 4, and IL-13, among others. This fact was not observed in our study cohort in C-section-delivered babies at one month of age.

Microbial exposure stimulates Ig production in infants, and Ig regulates the gut microbiota composition in crosstalk between the gut microbiota and immune system. In the present study, colonization via *Enterococcus*, *Blautia*, or *Bifidobacterium*, a normal member of the infant gut, was negatively associated with the levels of IgA and IgM in full-term infants; however, premature babies showed a positive correlation between IgA and the *Serratia* genus, which is a potential pathogenic enterobacterium.

This study was carried out in the context of a cohort of 67 infants that make up a comprehensive and representative dataset for studying the early faecal immune profile. Despite the limitations of posing only one sampling time-point and being unable to assess the progression and different immune maturation statuses of the gut, the results presented here suggest that factors affecting the early gut microbiota are also associated with early immune development.

## 4. Conclusions

Overall, the findings of this study reinforce the hypothesis that microorganisms and immune compounds interact with each other in the early neonatal gut. Full-term and premature infants showed that different immune profiling and factors affecting the early gut microbiota are also associated with early immune maturation. Understanding these interactions in depth will help us understand the influence of the gut microbiota on short- and long-term infant health outcomes.

## 5. Materials and Methods

### 5.1. Volunteers and Faecal Sample Collection

A total of 67 infants born at the Central University Hospital of Asturias (Oviedo, Northern Spain) were enrolled in this study. Thirty-seven neonates (19 females/18 males) were full-term (38–41 gestational weeks) and vaginally delivered. Twelve full-term mothers received intrapartum antimicrobial prophylaxis (IAP). None of the full-term infants received antibiotics nor had any early-onset infection or NEC. Twenty-five infants were breast-fed, and three received mixed feeding (infant formula and some breast milk) at one month of life. They were discharged from the hospital by their third day of life. A total of 30 neonates (18 females/12 males) were very-low-birthweight (1234.90 ± 274.49 g) preterm infants (24–35 gestational weeks), with 22 of them being delivered via C-section. Sixteen of the preterm infants’ mothers received IAP. Sixteen premature infants received antibiotics during the first two weeks of life but none of them developed NEC. Ten premature infants received the formula milk regimen, while the rest of the one-month preterm neonates had mixed feeding. They were discharged from the hospital after an average hospital stay of 50 (range 21–93) days. Exclusion criteria were as follows: the use of antibiotics, probiotics, or prebiotics in the month prior to stool collection. None of the mothers received antibiotics during pregnancy or the postnatal period, other than the abovementioned.

At the age of 30 days, a fresh faecal sample was collected in a sterile container by the full-term infant’s parents or by a paediatrician in the case of premature infants. Samples were immediately frozen at −20 °C until further analyses.

This study was approved by the Regional Ethical Committee of Asturias Public Health Service (SESPA). Individual informed signed consent forms were obtained from each infant’s parents before participating in this study. This study was carried out in accordance with the Declaration of Helsinki.

### 5.2. Faecal Sample Processing

At the lab and under sterile conditions, faecal samples were processed after being allowed to thaw on ice. A total of 1 g of a faecal sample was diluted in a 1:10 (*w*/*v*) ratio in a sterile PBS solution and homogenized in a LabBlender 400 stomacher (Seward Medical, London, UK) at full speed for 3 min. A total of 1 mL of the homogenized sample was centrifuged for 15 min at full speed and 4 °C. After sedimentation, cell pellets were kept at −20 °C for microbial DNA isolation, and faecal water was aliquoted in different tubes for the subsequent immunological and metabolite analyses and kept at −20 °C.

### 5.3. Faecal Immune Compound Analyses

Faecal water was used to determine the concentration of 15 immune factors, including immunoglobulins and cytokines related to Th1/Th2 balance—according to the manufacturer’s protocol—using magnetic bead-based multiplex immunoassays, Bio-Plex Pro Human Isotyping, and Bio-Plex Pro Human Cytokine Assays (Bio-Rad), respectively, in a Bio-Plex MAGPIX Multiplex Reader (Bio-Rad, Hercules, CA, USA). Data acquisition was carried out with the Bio-Plex Manager 6.0 software, and the standard curves were fitted to a 5-parameter logistic regression. The concentrations of the immune compounds were expressed as ng/g of faeces for IgG1, IgG3, and IgG4; µg/g of faces for IgG2 and IgM; mg/g of faeces for IgA; and pg/g of faeces for cytokines.

### 5.4. Gut Microbiota Analyses

DNA was isolated from faecal cell pellets following the Qiagen manufacturer’s instructions (QIAmp DNA stool kit, Qiagen GmbH, Hilden, Germany). The region V3-V4 of the 16S rRNA gene was amplified according to previous protocols [33] and sequenced using the MiSeq (Illumina, San Diego, CA, USA) platform at GenProbio s.r.l. (Parma, Italy). The individual reads obtained were filtered, trimmed, and processed, and 16S rRNA Operational Taxonomic Units were defined at ≥97% sequence homology and classified into the lowest possible taxonomic rank using QIIME and a reference dataset from the SILVA database, as described previously [34].

A chromatographic system composed of a 6890NGC injection module connected to a flame injection detector (FID) and a mass spectrometry detector (MS, 5973N) (Agilent Technologies, Madrid, Spain) was used to determine the concentration of short- and branched-chain fatty acids in the faecal water supernatants of each sample, as previously defined [34]. The concentrations of the analysed metabolites were calculated in millimolar (mM) and referred to as concentrations in the faecal samples.

### 5.5. Statistical Analyses

Immune faecal data were analysed using the software SPSS v.26 (SPSS Inc., Chicago, IL, USA). Normality was checked using the Shapiro–Wilk test. As the variables were not normally distributed, medians and interquartile ranges (P1 and P3) were calculated, and comparisons were performed using the non-parametric Mann–Whitney or Kruskal–Wallis test (plus Dunn’s test for pairwise comparisons). Pearson’s χ2 test was used to assess the occurrence of the different immune compounds analysed among groups. Finally, Spearman’s correlation method was conducted by using the rcorr() function [in the Hmisc R-package] to elucidate the relationship between the microbial and immune variables. The level of significance was set at a *p* value of <0.05.

## Figures and Tables

**Table 1:** The occurrence (%) of the different immune compounds analysed in faecal samples from one-month-old full-term (*n* = 37) and premature (*n* = 30) infants.

Infant Group		Full-Term	Premature	*p* Value
Immune Compounds				
Immunoglobulins (%)				
	IgG1	89.19	80.00	0.294
	IgG2	100.00	100.00	1.000
	IgG3	35.14	6.67	0.005
	IgG4	94.59	70.00	0.007
	IgA	100.00	93.33	0.111
	IgM	100.00	96.67	0.263
Cytokines (%)				
	IL-2	0.00	40.00	0.000
	IL-4	24.32	60.00	0.003
	IL-5	2.70	43.33	0.000
	IL-10	0.00	40.00	0.000
	IL-12 (p70)	37.84	60.00	0.071
	IL-13	0.00	40.00	0.000
	GM-CSF	0.00	36.67	0.000
	IFN-γ	0.00	46.67	0.000
	TNF-α	0.00	40.00	0.000

%: percentage of positive samples.

**Table 2:** Concentration (median—IQR) of different cytokines analysed in faecal samples from full-term (*n* = 37) and premature (*n* = 30) 1-month-old infants.

Cytokines (pg/g)	Full-Term	Premature	*p* Value
IL-2	14.40 (14.40–14.40)	14.40 (14.40–27.93)	<0.001
IL-4	1.20 (1.20–2.02)	2.83 (1.20–17.67)	<0.001
IL-5	40.30 (40.30–40.30)	40.30 (40.30–97.67)	<0.001
IL-10	17.10 (17.10–17.10)	17.10 (17.10–31.04)	<0.001
IL-12 (p70)	3.97 (3.97–3.97)	3.97 (3.97–21.62)	<0.001
IL-13	5.60 (5.60–5.60)	5.60 (5.60–11.07)	<0.001
GM-CSF	5.30 (5.30–5.30)	5.30 (5.30–5.93)	<0.001
IFN-γ	12.68 (12.68–12.68)	12.68 (12.68–35.90)	<0.001
TNF-α	40.20 (40.20–40.20)	40.20 (40.20–81.22)	<0.001

**Table 3:** Concentration (median—IQR) of immunoglobulins in faecal samples from one-month-old full-term breast-fed (*n* = 25), formula-fed (*n* = 9), and mixed-fed (*n* = 3) infants and one-month-old premature formula-fed (*n* = 10) and mixed-fed (*n* = 19) infants.

Infant Group		Breast-Fed	Formula-Fed	Mixed-Fed	*p* Value
Immunoglobulins					
Full-term					
	IgG1 (ng/g)	417.47 (230.17–912.98)	1657.25 (178.98–4145.41)	116.41 (57.5–1653.93)	0.251
	IgG2 (µg/g)	68.79 (38.94–109.68)	26.03 (12.87–50.61)	22.11 (21.25–23.34)	0.004
	IgG3 (ng/g)	31.25 (31.25–49.49)	31.25 (31.25–418.70)	31.25 (31.25–80.36)	0.939
	IgG4 (ng/g)	28.72 (13.19–44.48)	11.03 (7.03–15.32)	5.74 (5.46–18.99)	0.014
	IgA (mg/g)	7.45 (4.21–13.10)	1.67 (0.35–2.65)	1.54 (0.69–9.79)	0.001
	IgM (µg/g)	25.78 (14.2–61.86)	17.91 (1.84–25.64)	20.75 (17.34–71.31)	0.269
Premature					
	IgG1 (ng/g)	–	401.15 (65.35–610.46)	754.19 (195.45–1401.51)	0.330
	IgG2 (µg/g)	–	8.41 (3.36–15.20)	24.43 (4.54–46.20)	0.045
	IgG3 (ng/g)	–	31.25 (31.25–31.25)	31.25 (31.25–31.25)	0.668
	IgG4 (ng/g)	–	5 (5–21.67)	28.01 (14.08–105.3)	0.021
	IgA (mg/g)	–	0.04 (0.02–0.24)	2.72 (0.19–6.79)	0.001
	IgM (µg/g)	–	4.24 (0.41–11.74)	9.86 (3.45–53.15)	0.077

## Data Availability

Additional data presented in this study are available upon reasonable request from the corresponding author.

## Supplementary Materials

The following supporting information can be downloaded at https://www.mdpi.com/article/10.3390/ijms251910675/s1.

## References

[1] Zheng D., Liwinski T., Elinav E. (2020). Interaction between microbiota and immunity in health and disease. Cell Res..

[2] Olszak T., An D., Zeissig S., Vera M.P., Richter J., Franke A., Glickman J.N., Siebert R., Baron R.M., Kasper D.L. (2012). Microbial exposure during early life has persistent effects on natural killer T cell function. Science.

[3] Milani C., Duranti S., Bottacini F., Casey E., Turroni F., Mahony J., Belzer C., Delgado Palacio S., Arboleya Montes S., Mancabelli L. (2017). The First Microbial Colonizers of the Human Gut: Composition, Activities, and Health Implications of the Infant Gut Microbiota. Microbiol. Mol. Biol. Rev..

[4] Battersby A.J., Gibbons D.L. (2013). The gut mucosal immune system in the neonatal period. Pediatr. Allergy Immunol..

[5] Kalbermatter C., Fernandez Trigo N., Christensen S., Ganal-Vonarburg S.C. (2021). Maternal Microbiota, Early Life Colonization and Breast Milk Drive Immune Development in the Newborn. Front. Immunol..

[6] Zachariassen L.F., Krych L., Rasmussen S.H., Nielsen D.S., Kot W., Holm T.L., Hansen A.K., Hansen C.H.F. (2019). Cesarean Section Induces Microbiota-Regulated Immune Disturbances in C57BL/6 Mice. J. Immunol..

[7] Zachariassen L.F., Hansen A.K., Krych L., Nielsen D.S., Holm T.L., Tougaard P., Hansen C.H.F. (2019). Cesarean Section Increases Sensitivity to Oxazolone-Induced Colitis in C57BL/6 Mice. Mucosal Immunol..

[8] Andersen V., Möller S., Jensen P.B., Møller F.T., Green A. (2020). Caesarean Delivery and Risk of Chronic Inflammatory Diseases (Inflammatory Bowel Disease, Rheumatoid Arthritis, Coeliac Disease, and Diabetes Mellitus): A Population Based Registry Study of 2,699,479 Births in Denmark During 1973–2016. Clin. Epidemiol..

[9] Stokholm J., Thorsen J., Blaser M.J., Rasmussen M.A., Hjelmsø M., Shah S., Christensen E.D., Chawes B.L., Bønnelykke K., Brix S. (2020). Delivery Mode and Gut Microbial Changes Correlate with an Increased Risk of Childhood Asthma. Sci. Trans. Med..

[10] Tosi M., Coloretti I., Meschiari M., De Biasi S., Girardis M., Busani S. (2024). The Interplay between Antibiotics and the Host Immune Response in Sepsis: From Basic Mechanisms to Clinical Considerations: A Comprehensive Narrative Review. Antibiotics.

[11] Zhang X., Borbet T.C., Fallegger A., Wipperman M.F., Blaser M.J., Müller A. (2021). An Antibiotic-Impacted Microbiota Compromises the Development of Colonic Regulatory T Cells and Predisposes to Dysregulated Immune Responses. mBio.

[12] Wood H., Acharjee A., Pearce H., Quraishi M.N., Powell R., Rossiter A., Beggs A., Ewer A., Moss P., Toldi G. (2021). Breastfeeding Promotes Early Neonatal Regulatory T-cell Expansion and Immune Tolerance of non-Inherited Maternal Antigens. Allergy.

[13] Gopalakrishna K.P., Macadangdang B.R., Rogers M.B., Tometich J.T., Firek B.A., Baker R., Ji J., Burr A.H., Ma C., Good M. (2019). Maternal IgA Protects Against the Development of Necrotizing Enterocolitis in Preterm Infants. Nat. Med..

[14] Brenmoehl J., Ohde D., Wirthgen E., Hoeflich A. (2018). Cytokines in Milk and the Role of TGF-Beta. Best. Pract. Res. Clin. Endocrinol. Metab..

[15] Gómez M., Moles L., Espinosa-Martos I., Bustos G., de Vos W.M., Fernández L., Rodríguez J.M., Fuentes S., Jiménez E. (2017). Bacteriological and Immunological Profiling of Meconium and Fecal Samples from Preterm Infants: A Two-Year Follow-Up Study. Nutrients.

[16] Corthesy B. (2013). Multi-faceted functions of secretory IgA at mucosal surfaces. Front. Immunol..

[17] Kubinak J.L., Petersen C., Stephens W.Z., Soto R., Bake E., O’Connell R.M., Round J.L. (2015). MyD88signaling in T cells directs IgA-mediated control of the microbiota to promote health. Cell Host Microbe.

[18] Dzidic M., Abrahamsson T.R., Artacho A., Björkstén B., Collado M.C., Mira A., Jenmalm M.C. (2017). Aberrant IgA responses to the gut microbiota during infancy precede asthma and allergy development. J. Allergy Clin. Immunol..

[19] Kany S., Vollrath J.T., Relja B. (2019). Cytokines in Inflammatory Disease. Int. J. Mol. Sci..

[20] Khan S.R., van der Burgh A.C., Peeters R.P., van Hagen P.M., Dalm V.A.S.H., Chaker L. (2021). Determinants of Serum Immunoglobulin Levels: A Systematic Review and Meta-Analysis. Front. Immunol..

[21] Kim H.O., Kim H.S., Youn J.C., Shin E.C., Park S. (2011). Serum cytokine profiles in healthy young and elderly population assessed using multiplexed bead-based immunoassays. J. Transl. Med..

[22] Kleiner G., Marcuzzi A., Zanin V., Monasta L., Zauli G. (2013). Cytokine levels in the serum of healthy subjects. Mediat. Inflamm..

[23] Guadamuro L., Diaz M., Jiménez S., Molinos-Norniella C., Pérez-Solis D., Rodríguez J.M., Bousoño C., Gueimonde M., Margolles A., Delgado S. (2019). Fecal Changes Following Introduction of Milk in Infants With Outgrowing Non-IgE Cow’s Milk Protein Allergy Are Influenced by Previous Consumption of the Probiotic LGG. Front. Immunol..

[24] Castro I., García-Carral C., Furst A., Khwajazada S., García J., Arroyo R., Ruiz L., Rodríguez J.M., Bode L., Fernández L. (2022). Interactions between human milk oligosaccharides, microbiota and immune factors in milk of women with and without mastitis. Sci. Rep..

[25] Neu J. (2015). Developmental aspects of maternal-fetal, and infant gut microbiota and implications for long-term health. Matern. Health Neonatol. Perinatol..

[26] Stiemsma L.T., Michels K.B. (2018). The Role of the Microbiome in the Developmental Origins of Health and Disease. Pediatrics.

[27] Suzuki K., Ha S., Tsuji M., Fagarasan S. (2007). Intestinal IgA synthesis: A primitive form of adaptive immunity that regulates microbial communities in the gut. Semin. Immunol..

[28] Bridgman S.L., Konya T., Azad M.B., Sears M.R., Becker A.B., Turvey S.E., Mandhane P.J., Subbarao P., Scott J.A., CHILD Study Investigators (2016). Infant gut immunity: A preliminary study of IgA associations with breastfeeding. J. Dev. Orig. Health Dis..

[29] Maruyama K., Hida M., Kohgo T., Fukunaga Y. (2009). Changes in salivary and fecal secretory IgA in infants under different feeding regimens. Pediatr. Int..

[30] Granger C.L., Lamb C.A., Embleton N.D., Beck L.C., Masi A.C., Palmer J.M., Stewart C.J., Berrington J.E. (2022). Secretory immunoglobulin A in preterm infants: Determination of normal values in breast milk and stool. Pediatr. Res..

[31] Chen Y.Y., Tun H.M., Field C.J., Mandhane P.J., Moraes T.J., Simons E., Turvey S.E., Subbarao P., Scott J.A., Kozyrskyj A.L. (2023). Impact of Cesarean Delivery and Breastfeeding on Secretory Immunoglobulin A in the Infant Gut Is Mediated by Gut Microbiota and Metabolites. Metabolites..

[32] Hocke A.C., Ermert M., Althoff A., Brell B., N’Guessan P.D., Suttorp N., Ermert L. (2006). Regulation of interleukin IL-4, IL-13, IL-10, and their downstream components in lipopolysaccharide-exposed rat lungs. Comparison of the constitutive expression between rats and humans. Cytokine.

[33] Milani C., Hevia A., Foroni E., Duranti S., Turroni F., Lugli G.A., Sanchez B., Martin R., Gueimonde M., van Sinderen D. (2013). Assessing the fecal microbiota: An optimized ion torrent 16S rRNA gene-based analysis protocol. PLoS ONE.

[34] Higarza S.G., Arboleya S., Gueimonde M., Gómez-Lázaro E., Arias J.L., Arias N. (2019). Neurobehavioral dysfunction in non-alcoholic steatohepatitis is associated with hyperammonemia, gut dysbiosis, and metabolic and functional brain regional deficits. PLoS ONE.

