# Supplementary material for: Impacts of Different Perinatal Factors on Faecal Immune Compounds in Infants: Determination of Normal Values
Source: Int J Mol Sci. 2024 Oct 3;25(19):10675. doi: 10.3390/ijms251910675 (PMC11477395; doi:10.3390/ijms251910675)
Supplement: Supplementary file 1 [file ijms-25-10675-s001.zip › ijms-3162999-supplementary.pdf]

Table S1: Occurrence (% of positive samples) of the different immune-compounds analyzed in fecal samples from full-term breast-fed (n = 25), formula-fed (n = 9) and mixed-fed (n = 3); and premature formula-fed (n=10) and mixed-fed (n=19) one-month-old infants.

**Infant group** / **Immune-compounds**	**Breast-fed**	**Formula-fed**	**Mixed-fed**	***p. value***
**FULL-TERM**				
**Immunoglobulins (%)**				
**IgG1**	92.00	88.89	66.67	0.410
**IgG2**	100.00	100.00	100.00	n.s.
**IgG3**	36.00	33.33	33.33	0.987
**IgG4**	96.00	88.89	100.00	0.657
**IgA**	100.00	100.00	100.00	n.s.
**IgM**	100.00	100.00	100.00	n.s.
**Cytokines (%)**				
**IL-2**	0.00	0.00	0.00	n.s.
**IL-4**	20.00	44.44	0.00	0.202
**IL-5**	0.00	0.00	33.33	**0.003**
**IL-10**	0.00	0.00	0.00	n.s.
**IL-12(p70)**	32.00	55.56	33.33	0.452
**IL-13**	0.00	0.00	0.00	n.s.
**GM-CSF**	0.00	0.00	0.00	n.s.
**IFN γ**	0.00	0.00	0.00	n.s.
**TNFα**	0.00	0.00	0.00	n.s.
**PREMATURE**				
**Immunoglobulins (%)**				
**IgG1**	-	80.00	78.95	0.877
**IgG2**	-	100.00	100.00	n.s.
**IgG3**	-	0.00	10.53	0.538
**IgG4**	-	40.00	84.21	**0.038**
**IgA**	-	80.00	100.00	0.117
**IgM**	-	100.00	94.74	0.741
**Cytokines (%)**	-			
**IL-2**	-	70.00	26.32	*0.052*
**IL-4**	-	70.00	57.89	0.377
**IL-5**	-	70.00	31.58	0.094
**IL-10**	-	70.00	26.32	*0.052*
**IL-12(p70)**	-	70.00	57.89	0.377
**IL-13**	-	70.00	26.32	*0.052*
**GM-CSF**	-	60.00	26.32	0.150
**IFN γ**	-	70.00	36.84	0.150
**TNFα**	-	70.00	26.32	*0.052*

n.s.: no statistical differences

Table S2: Concentration (median - IQR) of immunoglobulins in fecal samples from full-term no-IAP (n = 25) and IAP (n = 12); and premature formula-fed no-IAP (n = 14) and IAP (n = 16) one-month-old infants.

Infant group<br>Immunoglobulins	NO-IAP	IAP	*p. value*
**FULL-TERM**			
**IgG1 (ng/g)**	396.47 (167.32 - 699.67)	1415.4 (310.09 - 4203.08)	**0.038**
**IgG2 (µg/g)**	47.83 (24.60 - 84.86)	75.30 (26.47 - 128.82)	0.227
**IgG3 (ng/g)**	31.25 (31.25 - 56.84)	31.25 (31.25 - 56.15)	0.666
**IgG4 (ng/g)**	16.06 (9.1 - 33.07)	37.03 (13.79 - 76.03)	*0.061*
**IgA (mg/g)**	5.03 (1.61 - 11.96)	4.71 (2.32 - 10.16)	1.000
**IgM (µg/g)**	24.73 (13.81 - 33.41)	63.86 (13.96 - 171.22)	0.133
**PREMATURE**			
**IgG1 (ng/g)**	520.03 (57.5 - 1107.36)	408.15 (287.01 - 1311.14)	0.790
**IgG2 (µg/g)**	19.00 (3.88 - 33.20)	14.55 (4.69 - 37.58)	0.886
**IgG3 (ng/g)**	31.25 (31.25 - 31.25)	31.25 (31.25 - 31.25)	0.580
**IgG4 (ng/g)**	22.10 (7.58 - 52.04)	17.64 (5.00 - 123.78)	0.918
**IgA (mg/g)**	0.11 (0.03 - 4.94)	0.77 (0.08 - 4.66)	0.275
**IgM (µg/g)**	4.08 (1.10 - 49.43)	9.77 (1.60 - 52.38)	0.608

IQR: interquartile range

Table S3: Concentration (median - IQR) of immunoglobulins in fecal samples from full-term female (n = 19) and male (n = 18); and premature female (n = 18) and male (n = 12) one-month-old infants.

Infant group<br>Immunoglobulins	Female	Male	*p. value*
**FULL-TERM**			
**IgG1 (ng/g)**	450.04 (141.8 - 1348.82)	434.68 (213.57 - 1668.89)	0.845
**IgG2 (µg/g)**	45.86 (23.34 - 98.96)	65.07 (33.23 - 119.42.)	0.178
**IgG3 (ng/g)**	31.25 (31.25 - 49.2)	31.25 (31.25 - 59.82)	0.620
**IgG4 (ng/g)**	11.30 (5.74 - 32.55)	30.35 (17.89 - 55.76)	**0.007**
**IgA (mg/g)**	4.04 (1.39 - 13.17)	5.36 (2.07 - 8.85)	1.000
**IgM (µg/g)**	17.91 (8.86 - 25.78)	38.10 (20.92 - 90.69)	**0.007**
**PREMATURE**			
**IgG1 (ng/g)**	474.94 (233.81 - 1861.17)	380.86 (57.5 - 1006.61)	0.305
**IgG2 (µg/g)**	14.55 (4.86 - 32.55)	13.38 (3.84 - 40.11)	0.692
**IgG3 (ng/g)**	31.25 (31.25 - 31.25)	31.25 (31.25 - 31.25)	0.632
**IgG4 (ng/g)**	23.74 (7.58 - 94.01)	14.95 (5.00 - 89.44)	0.465
**IgA (mg/g)**	0.17 (0.05 - 3.61)	0.83 (0.04 - 6.09)	0.491
**IgM (µg/g)**	10.53 (1.10 - 51.78)	3.88 (0.62 - 42.07)	0.232

IQR: interquartile range

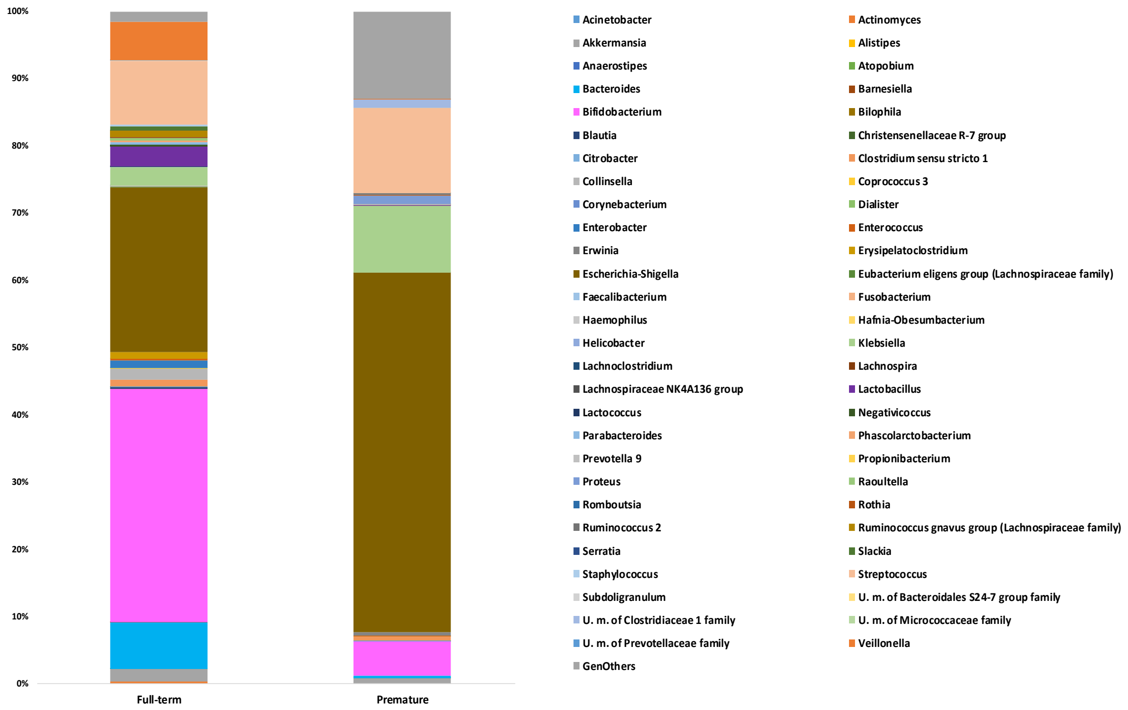

Figure S1: Aggregate microbiota composition at the family level (relative abundance) in fecal samples from one-month old full-term (n = 37) and premature (n = 30) infants.

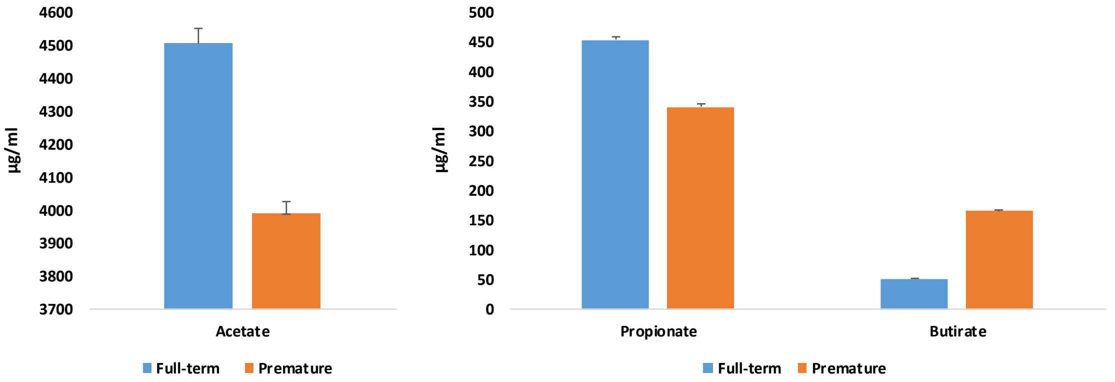

Figure S2: Concentration (µg/ml) of main short-chain fatty acids in fecal samples from one-month old full-term (n = 37) and premature (n = 30) infants.
